# Revealing The Morphology of Ink and Aerosol Jet Printed Palladium‐Silver Alloys Fabricated from Metal Organic Decomposition Inks

**DOI:** 10.1002/advs.202306561

**Published:** 2023-12-25

**Authors:** Nicholas T.H Farr, Matthew Davies, James Nohl, Kerry J. Abrams, Jan Schäfer, Yufeng Lai, Torsten Gerling, Nicola Stehling, Danielle Mehta, Jingqiong Zhang, Lyudmila Mihaylova, Jon R. Willmott, Kate Black, Cornelia Rodenburg

**Affiliations:** ^1^ Department of Materials Science and Engineering Sir Robert Hadfield Building Mappin Street University of Sheffield Sheffield S1 3JD UK; ^2^ Insigneo Institute for In Silico Medicine, The Pam Liversidge Building Sheffield S1 3JD UK; ^3^ Department of Electronic and Electrical Engineering Portobello Centre, Pitt Street. University of Sheffield Sheffield S1 4ET UK; ^4^ Leibniz Institute for Plasma Science and Technology e.V. (INP) Felix‐Hausdorff‐Str. 2 17489 Greifswald Germany; ^5^ School of Engineering University of Liverpool Liverpool L69 3BX UK; ^6^ Department of Automatic Control and Systems Engineering The University of Sheffield Amy Johnson Building, Portobello Street Sheffield S1 3JD UK

**Keywords:** aerosoljet printing, inkjet printing, metal organic decomposition inks, palladium‐silver alloys, plasma modification

## Abstract

Palladium films hold signicance due to their remarkable affinity for hydrogen diffusion, rendering them valauble for the seperation and purification of hydrogen in membrane reactors. However, palladium is expensive, and its films can become brittle after only a few cycles of hydrogen separation. Alloying with silver has been shown to overcome the problem of palladium embrittlement. Palladium‐silver films have been produced via several methods but all have drawbacks, such as difficulties controlling the alloy composition. This study explores two promising jet printing methods: Inkjet and Aerosoljet. Both methods offer potential advantages such as direct patterning, which reduces waste, enables thin film production, and allows for the control of alloy composition. For the first time, palladium‐silver alloys have been produced via inkjet printing using a palladium‐silver metal organic decomposition (MOD) ink, which alloys at a temperature of 300 °C with nitrogen. Similarly, this study also demonstrates a pioneering approach for Aerosol Jet printing, showing the potential of a novel room‐temperature method, for the deposition of palladium‐silver MOD inks. This low temperature approach is considered an important development as palladium‐silver MOD inks are originally designed for deposition on heated substrates.

## Graphical abstract

For the first time, palladium‐silver alloys are produced via Inkjet and Aerosol‐Jet printing using the same MOD inks. A pioneering approach for room‐temperature deposition and annealing of Aerosol Jet printed MOD inks is demonstrated. Insights to localized nanoscale variation of film morphology and composition at the nanoscale, unobtainable through conventional methods, are enabled by Secondary Electron Hyperspectral Imaging.

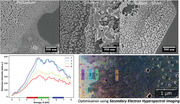

## 1 Introduction

Palladium membranes are widely used for hydrogen separation in catalytic membrane reactors, performing both the hydrogenation and dehydrogenation reactions.^[^ ^1^, ^2^ ^]^ Palladium is known to have unique properties including high hydrogen solubility (600‐900 times its gaseous volume at room temperature) which enables its use in hydrogen separation and purification.^[^ ^1^, ^2^, ^3^, ^4^ ^]^ Pure palladium films are expensive and have known limitations, such as palladium embrittlement occurring after a few separation cycles.^[^ ^1^, ^5^ ^]^ Temperature cycling of palladium membranes for the process of hydrogen purification leads to phase changes from an α phase to a β phase below 300 °C. The β phase lattice is 3% larger than the α phase. Consequently, repeated hydrogen purification can introduce lattice strain, resulting in embrittlement.^[^ ^6^ ^]^ One strategy to mitigate palladium embrittlement involves alloying it with other metals.^[^ ^7^ ^]^ Specifically, alloying with silver has been shown to reduce embrittlement whilst maintaining the affinity for hydrogen separation.^[^ ^2^ ^]^ The ratio in which palladium and silver are alloyed affects the film's ability to process hydrogen, with a ratio of around 77:23 palladium to silver being shown as optimum ratio for hydrogen separation.^[^ ^1^, ^2^ ^]^

Membranes of palladium‐silver alloys have previously been produced via several methods including: chemical and physical vapour deposition (CVD and PVD) ,^[^ ^2^, ^8^ ^]^ pyrolysis,^[^ ^2^, ^9^ ^]^ sputtering,^[^ ^2^, ^3^ ^]^ and electroless plating.^[^ ^2^, ^8^ ^]^ Electroless plating and CVD processes have the advantages of scalability and both processes are able to coat structures of varying geometries with the alloy.^[^ ^2^, ^4^, ^10^ ^]^ Sputtering allows for thin films to be produced and the ability to generate nanostructured films.^[^ ^2^, ^11^, ^12^ ^]^ However, a common drawback of these deposition techniques is their inability to uniformly control the alloys composition Moreover, they often produce non‐selective coatings during deposition.^[^ ^2^ ^]^ Conversely, inkjet printing is a low waste, non‐contact deposition technique and holds the promise to provide a viable alternative for manufacturing complex film geometries, while also controlling the composition of the deposited alloy.^[^ ^13^ ^]^ Aerosol jet printing is a more experimental, but potentially more flexible manufacturing process that allows for deposition of materials onto non‐planar substrates due to the greater working distance of the process. This process has already been applied in a diverse array of applications from electronic devices to radio‐frequency communications and microfluidics, in some instances deposited directly onto complex geometries.^[^ ^14^, ^15^, ^16^, ^17^ ^]^ Davies et al. demonstrated functioning a polymer dispersed liquid crystal device on a 90° apex of a prism.^[^ ^18^ ^]^ In this study two promising jet printing methods are applied, Ink and Aerosol. Both methods have the potential for direct patterning, a technique which reduces waste, enables thin film production, and could allow for control of the printed alloy composition. To the best of our knowledge, there are no existing examples of using either ink or aerosol jet printing for palladium‐silver alloys.

Inkjet printing alloys present problems because they require chemistries that react at similar post‐processing temperatures and dissolve in the same carrier vehicle, to produce uniform alloy films. Most commercial metal inks are particle‐based, which can introduce difficulties in printing, such as nozzle clogging and ink sedimentation.^[^ ^19^ ^]^ Given these challenges, there's a growing interest in solution‐based inks, such as MOD and reactive organometallic (ROM) inks, for inkjet printing.^[^ ^20^, ^21^ ^]^ Unlike particle‐based inks, solution‐based inks can be modified to adjust the stoichiometry of the metallic components when printing alloyed films. In this context, we introduced a palladium‐silver MOD ink designed to allow adjustments in the stoichiometry of metallic components as required. Inkjet printing of palladium and silver MOD inks have each been carried out showing that both metals are viable for printing.^[^ ^19^, ^21^, ^23^ ^]^ The current study demonstrates for the first time that alloy films can be obtained by both inkjet printing (on heated substrates followed by annealing in nitrogen) and a novel room‐temperature aerosol jet printing approach, which when followed by an innovative plasma jet annealing technique, has the capacity to be applied for the deposition of palladium‐silver MOD. By eliminating the need for high‐temperature annealing, aerosol jet printing onto temperature sensitive polymer substrates provides a new approach that would provide new insights on producing polymer substate Palladium‐Silver printed alloys. Recent studies have spotlighted these alloys as promising silver alloy catalysts for CO2 reduction in electrolyzer membranes.^[^ ^24^ ^]^ For such applications to be successful the alloy composition is critical. Using bulk averaging characterization films obtained via inkjet printing form MOD inks contain crystalline material with a molar ratio of 79:21 (palladium to silver) and exhibit complex morphologies across several length scales. Thus, Secondary Electron Hyperspectral Imaging (SEHI) was used to obtain novel insights, unobtainable through conventional methods, into the localized variation of the film morphology and alloy composition at the nanoscale. Using the same ink different jet‐printing and post printing approaches are explored to in the quest to obtaining homogeneous films.

## 2 Experimental Section

### 2.1 Materials and Equipment

Silver acetate and concentrated ammonium hydroxide were used as supplied by Sigma Aldrich. Palladium acetate was used as provided by Apollo Scientific Ltd. A palladium‐silver stock solution was made by mixing 3.547 g of palladium acetate with 0.701 g of silver acetate. To this powder mixture 15 mL of concentrated ammonium hydroxide was added dropwise over the course of an hour, whilst stirring using a magnetic stirrer bar. A MOD Pd‐Ag ink was made from the stock solution by mixing 1 mL of the stock solution with 4 mL of iso‐propanol (IPA).

### 2.2 Printing and Annealing Conditions

#### 2.2.1 Inkjet Printing

Printing was carried out using a MicroFab Jetlab 4 printing system in ambient atmosphere. The following waveform was identified for the PdAg MOD ink (**Table** **1**).

**Table 1:** Waveform used to generate droplet for Pd‐Ag MOD ink when using an 80 µm print head.

Rise Time (µs)	10
Dwell Time (µs)	32
Echo Time (µs)	64
Fall Time (µs)	10
Dwell Voltage (V)	32
Echo Voltage (V)	−32

Inks were printed with a drop spacing of 0.05 mm at a travelling velocity of 13 mm/s on glass at 60 °C. 1×1 cm films were produced and then heated to 300 °C for 1 hour and cooled in an inert nitrogen.

#### 2.2.2 Aerosoljet Printing

An Optomec AJ300 series aerosol jet deposition system was used to print the PdAg ink. The ink was diluted at a 1:7 weight ratio in isopropanol (PdAg:IPA) to facilitate atomization and 1 ml was loaded into the atomizer vial. The atomizer power was set to the maximum value (48 V at 650 mA). A nitrogen carrier gas was used and material was printed with a gas flow of 30 cm^3^min^−1^ and the focusing ratio (ratio of focusing gas flow and carrier gas flow) was 3. The ink was chilled to 10 °C and printed in ambient atmosphere onto pre‐existing PEDOT:PSS films on both glass and silicone substrates using a 150 µm nozzle. 4 squares were printed (50 µm, 100 µm, 500 µm, and 1000 µm) on each substrate using a perimeter infill pattern, with a 10 µm pitch, at a speed of 2 mms^−1^. Plasma technology was then used instead of heat treatment. Plasma jets,^[^ ^25^ ^]^ in this case Hairline treated the samples through which a similar effect to heating was achieved at the local level by the action of reactive non‐isothermal processes (e.g., argon metastables recombination, eximer dissociation). These processes are typical for plasma discharges under atmospheric pressure, creating a strong application potential for printing even in 3D dimensions^[^ ^25^ ^]^ at temperatures suitable for polymer substrates.

### 2.3 Characterization of Ink for Inkjet Printing

The ink's rheological properties were assessed in terms of viscosity, surface tension and contact angle. Viscosity measurements were carried out using a MicroVISC viscometer. Surface tension and contact angle measurements were carried out using a Kruss DSA 100. All rheometry measurements were taken at room temperature. Density of the ink was calculated from the mass and volume of the ink produced using the following equation:

(1) ρ=m/V

Where ρ was density, m was mass of ink and V was volume of ink.

### 2.4 Printed Film Characterization

#### 2.4.1 Microscopy

Optical images were taken using a Leitz Wetzlar Metalloplan microscope and a Canon 70D DSLR camera with a Canon Macro EF 100 mm lens. Standard Scanning electron microscopy (SEM) images were taken using a JEOL 6610 with an accelerating voltage of 10 kV. High resolution images were taken using Low (LV‐SEM) accelerating voltage of 1 kV and the Through‐the‐lens‐detector (TLD) in ultra high resolution mode at working distances between 3 mm – 4 mm in either FEI Nova Nano 450 or Helios Nanolab G3 UC.

#### 2.4.2 Chemical Analysis

Secondary Electron Hyperspectral Imaging (SEHI) was carried out in the FEI Nova Nano 450 SEM by controlling the TLD deflector electrode as described in^[^ ^26^, ^27^ ^]^ the tube bias voltage was 250 V, deflector voltage steps were 0.2 V, working distance 3 mm, accelerating voltage of 1 kV, with a scan interlace of 8 to minimize any sample charging. Spectra and hyperspectral images were acquired through post‐processing of the image series, as preformed in previous studies.^[^ ^28^, ^29^ ^]^ Characterization of the printed alloy was also undertaken using X‐ray diffraction (XRD), which was performed on a Rigaku Smartlab diffractometer, using Cu Kα radiation spanning a 2θ range of 30° to 70° at a scan rate of 0.01 °/min.

## 3 Results and Discussion

### 3.1 Assessment of Palladium‐Silver MOD Ink and Track Optimization for Inkjet Printing

#### 3.1.1 Rheology Properties of MOD Ink

The developed ink was assessed in terms of the rheological properties and the results obtained are presented in **Table** **2**.

**Table 2:** Rheological properties for Pd‐Ag MOD ink (properties may change in the print head due to shear forces in the nozzle) compared with that of known Pd and Ag properties.

Ink	Contact Angle [°]	Viscosity (cP)	Surface Tension [mN/m]	Density [g cm^−3^]	Oh	Z Number [1/Oh]
PdAg Ink	9.18 (glass)	5.58	19.25	0.85	0.15	6.48

Viscosity and surface tension were measured, so it could be identified if the ink was within the suitable ranges for inkjet printing. The Ohnesorge and Z number, which determines printability, were also calculated. From Table 2 it is apparent that the viscosity of the ink was within the required range (0—20 cP).^[^ ^30^ ^]^ However, the surface tension was 19.25 mN/m, which was 0.7 mN/m below the lower surface tension limit for the printer, of 20—70 mN/m.^[^ ^30^ ^]^ The Ohnesorge number is calculated from the following equation:

(2) $$Oh\;=\frac{\mu }{\surd \left(d\rho \gamma \right)}$$

Where µ is the viscosity, d is the nozzle diameter, γ is the surface tension and ρ is fluid density. Printability can be calculated from the inverse of the Ohnesorge. Literature has identified that the Z number should be in the range of 1–14.^[^ ^31^, ^32^ ^]^ From Table 2 it is evident that the Z number for the palladium‐silver ink, 6.48, is within this range, suggesting the ink should be suitable for inkjet printing. Surface tension and viscosity may vary from the measured values when printing, due to the printing process being carried out with the stage heated to 60 °C, causing the print head to be at an elevated temperature. All measurements were carried out at room temperature because the equipment used is not able to carry out measurements at elevated temperatures. Rheological properties may also vary due to the shear forces within the nozzle. The surface tension and viscosity varying in the print head could also lead to the Z number being different when printing. The contact angle was measured to identify how the ink and substrate interact. From Table 2 a low contact angle of 9.18° on glass is obtained, indicating the substrate and ink interaction has a high wettability.

#### 3.1.2 MOD Ink Track Optimization for Inkjet Printing

**Figure 1:**
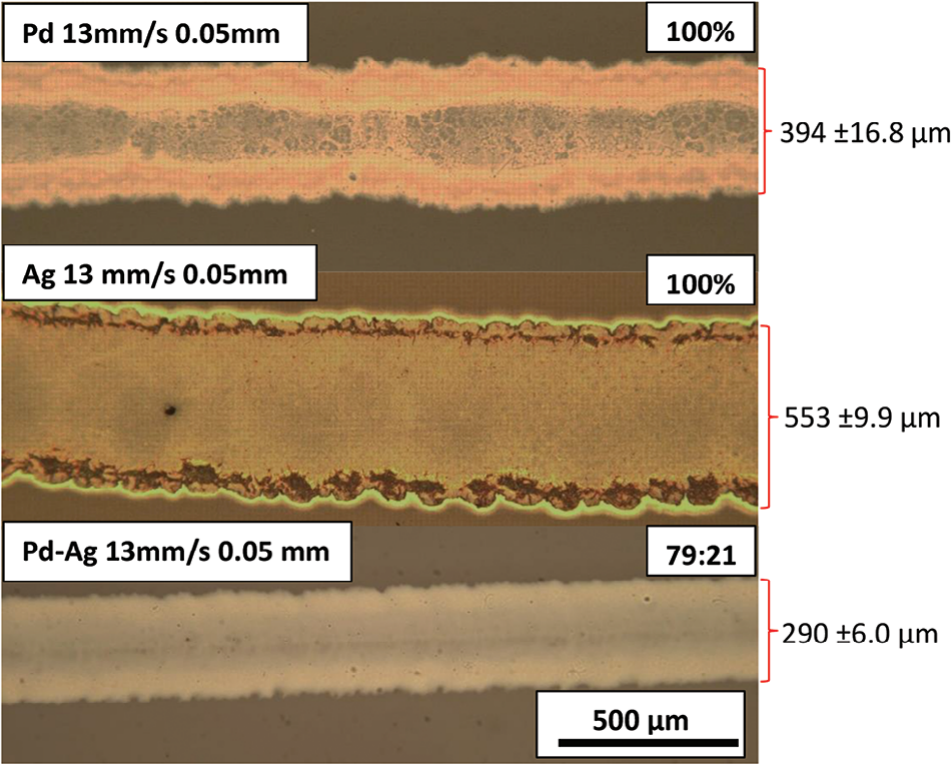
Optical images of tracks at x 80 magnification, printed using the optimum printing conditions of 13 mm/s travelling velocity and a 0.05 mm drop spacing on glass heated to 60 °C.

Once the ink had been identified as suitable for inkjet printing, optimization was carried out to identify the optimal printing conditions on glass. The tracks produced using the optimum conditions are shown in **Figure** **1**.

The inks tested were palladium, silver and a palladium‐silver MOD inks. All three inks were investigated so that the performance of palladium‐silver alloy films could be compared to pure palladium and pure silver films. Figure 1 presents the tracks produced with optimum parameters for each ink. All three inks produce continuous tracks with 0.05 mm drop spacing. It was apparent that the “ring effect” is present along the edge of all the optimized tracks, this effect is most prominent for the silver and palladium ink but the effect appears minimized when the alloy ink is used. The spreading of ink on the substrate, producing wide tracks at room temperature, is to be expected, based on the low contact angle obtained (Table 2).^[^ ^33^ ^]^

### 3.2 characterization of Inkjet Printed Films

#### 3.2.1 Assessment of Inkjet Film Morphology

**Figure 2:**
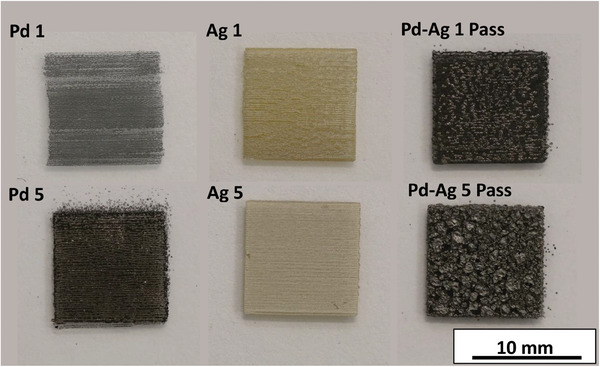
DSLR images of printed Pd, Ag, and Pd‐Ag printed films at one and five passes on glass after heat treatment has been carried out under nitrogen at 300 °C for 1 hour (glass substrate).

Palladium, silver and palladium‐silver alloy films were printed at one and five passes of the inkjet head. **Figure** **2** shows the films produced for the first and fifth passes of these inks. In general, all films produced have sharp well‐defined edges. However, when printing five passes, there is some spray evident around the edges of the films, this effect is most prominent for the palladium film. The spray may be due to secondary atomization occurring upon droplet impact. Secondary atomization may be occurring due to ammonia hydroxides boiling point (38 °C) being lower than the substrate temperature of 60 °C.^[^ ^34^, ^35^ ^]^

**Figure 3:**
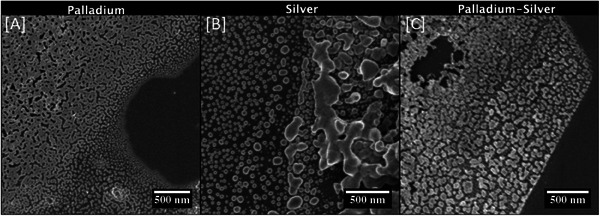
LV‐ SEM images of films deposited in one pass on silicon substrate.

When pure palladium films are produced it is apparent that there are inconsistencies present in the film at one pass. Individual lines can be distinguished, and reveal localized incomplete coverage, in areas where droplets have not spread to the same extent as in densely covered areas. The same effect, but less pronounced, is visible in the one pass silver film. Another observation worth noting is the color of the pass silver film. The film`s brown color is indicative of the presence of spherical nanoparticles within it.^[^ ^36^ ^]^ The one pass palladium‐silver film shows color variations that could either be as a result of thickness variations or local phase separation. To investigate these observations further, all of the above palladium‐silver films were also deposited on silicon substrates, in order to facilitate SEM imaging of any nanoparticles and their true size (charging of the glass substrate occurs in areas that are not connected to ground leading to image distortions). Low voltage SEM images for all three films deposited in pass are shown in **Figure** **3**.

In all films, the silicon substrate presents as a dark grey hue. Similar trends to the films deposited on glass (Figure 2) are observed. Specifically, large exposed regions are observed in the palladium film, whereas the silver film displays a more uniform coverage. The Palladium‐Silver film also exhibits incomplete coverage. In all three films shown in Figure 3 either 2D networks (in palladium containing films) or three dimensional (3D) agglomerations (in silver film), are visible in addition to individual spherical nanoparticles. In the palladium containing films these isolated spherical nanoparticles are only found near boarders between large uncovered areas of the silicon substrate and the film. The nanoparticles form 2D networks approximately a few hundred nanometers away from the border. In contrast, in the silver films, individual nanoparticles can be found several micrometers away from such boarder areas. We observe an overall reduction in size of these spherical nanoparticles the further away they are located from the 3D agglomerations.

**Figure 4:**
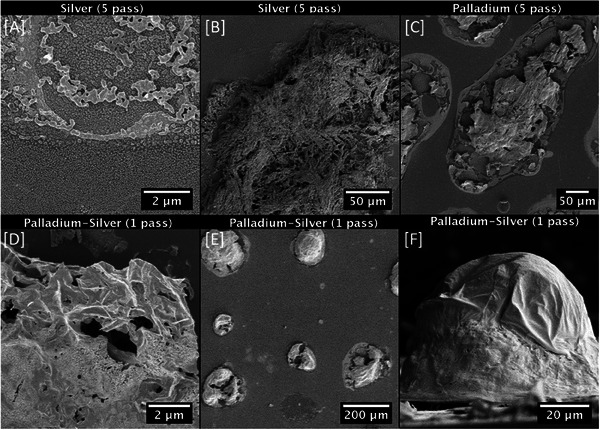
LV‐ SEM images of inkjet printed films deposited on silicon substrate showing droplet remnants.

These features are more obvious at lower magnification and are also present in the SE images obtained from the five‐pass silver film, in **Figure** **4**A. Figure 4A–D shows 3D agglomeration with a close to circular boundary and within this boundary. From Figure 4B it is apparent that these 3D agglomerates are remnants of former ink droplets that were not fully converted to silver nanoparticles during the annealing process, leaving behind porous 3D scaffoldlike structures in the silver films. Ink droplet remnants also exist in the five pass palladium films shown in Figure 4C. However, in palladium films, the remnants have a dense, sheet like appearance. Areas within the droplet, but no longer covered by such sheets, are covered by networks of palladium nanoparticles, similar to those shown in Figure 3C. This can be seen in Figure S1 (Supporting Information).

**Figure 5:**
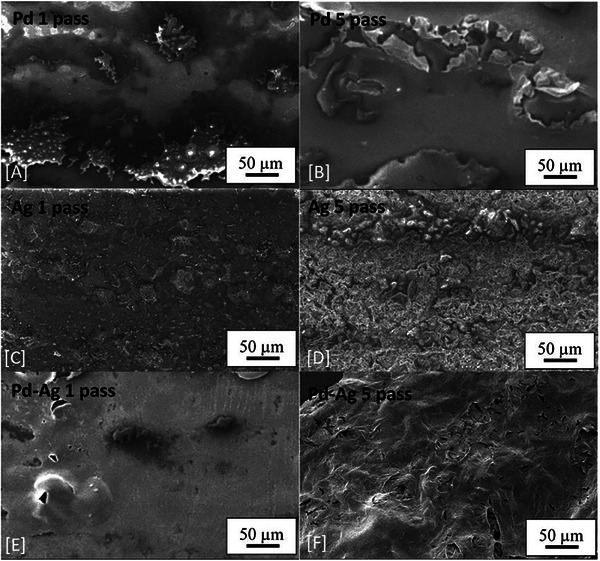
SEM images at x 270 magnification showing the microstructure of Pd, Ag, and Pd‐Ag printed films at one and five passes heat treated at 300 °C for 1 hour in nitrogen atmosphere (glass substrate).

Droplet remnants of palladium‐silver films exhibit characteristics of both the sheet‐like structures as observed in the palladium films (Figure 4C) and the more open, porous areas as observed in silver films (Figure 4B). These sheet‐like structures are shown on the example of the droplet remnants in a one pass palladium‐silver film in Figure 4D. Lower magnification image (Figure 4E) reveals the wide variation in size of such remnants and differences in the degree of porosity/presence of sheet structures. Extensive portions of these remnants can be covered by a single sheet, this is observable in the cross‐section displayed in Figure 4F. If shrinkage in the sheet structures have occurred, this can be deduced from the presence of folds in their structure. Such shrinkage can lead to partial collapse of the sheet feature, especially where there are larger droplet remnants. This partial collapse of the sheet structures is shown within the cross‐sectional images presented in Figure S2 (Supporting Information). This is notable as a common occurrence on the samples and can be seen in Figure 4E. The observed features on the silicon substrates are also seen on films inkjet printed on glass substrates. This observation explains the appearance of those films in the low magnification SEM images shown in **Figure** **5**.

In Figure 5 the one pass palladium film is observed to be discontinuous. Well defined makings are present in the one pass films that lead to the creation of similar sheet structures as seen in the five‐pass palladium film (that have partially collapsed – Figure 5 top right). The sheet structures are similar in size to those observed on silicon substrates (Figure 4). It is noted that the collapsed sheet structures are responsible for the micronscale surface variation of the palladium containing films. Smooth nanoparticle covered areas surround these sheet structures in the films deposited on silicon (Figure S1, Supporting Information) and glass substrates (Figure 5). Although the nanoparticles cannot be individually resolved in Figure 5, the local rupture of the sheet is observed on films deposited on both silicon (Figure 4B) and glass substrates (Figure 5). Figure 5 (bottom) further demonstrates that this effect prevails regardless of the number of passes. The 3D porous agglomerates observed on the silver films deposited on silicon substrates are also present in the silver films inkjet printed on glass substrates (Figure 5 center). One difference does exist within the boundaries of the palladium/silver alloy films deposited on silicon. These structures can be extremely well defined, as can be seen in Figure 3C. Such well‐defined geometries appear to be related to nanoparticles within the film (see Figure S3, Supporting Information).

In summary, the morphology of inkjet‐printed palladium/ silver alloy film is dominated by sheet structures on the micron scale that have the tendency to collapse or rupture, either due to the shrinkage or escape of volatile species during the thermal annealing treatment. Thus, it seems unlikely that the considerable surface variation of the palladium silver films could be eliminated by further optimization of the inkjet printing + thermal annealing process, unless a reduction in droplet size or increased wetting could be achieved. However, for where largely hollow micron‐scale structures are desirable, inkjet printing is considered suitable. Nonetheless, the question then arises as to what the chemical composition of these highly complex films are.

#### 3.2.2 Chemical Characterization of Inkjet Films

**Figure 6:**
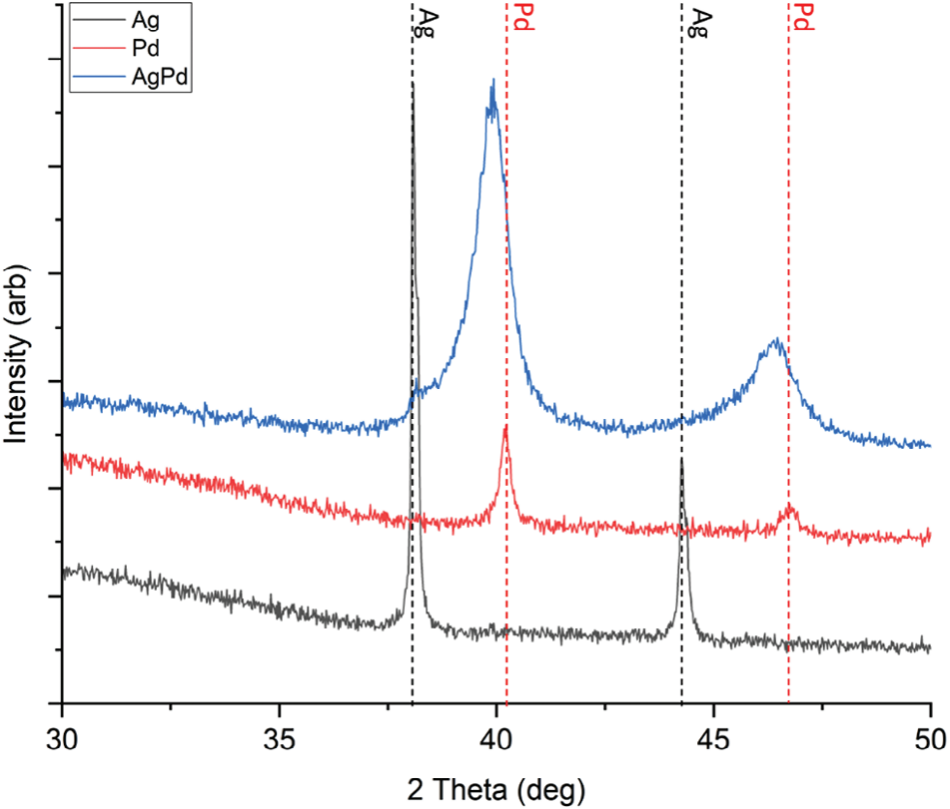
XRD of Pd, Ag, and Pd‐Ag films printed onto glass and heat treated at 300 °C for 1 hour in a nitrogen atmosphere.

The crystalline material within the alloy‐ and Silver‐, and Palladium‐ films that were deposited from MOD inks in 10 passes are can be assessed through the XRD signatures shown in **Figure** **6**. Palladium and Silver films deposited by one and five pass inkjet printing were too thin to be assessed by XRD. For the Silver and Palladium reference films all measured peaks were within 0.3° of the reference values, showing crystalline regions of pure palladium and silver films have been printed.^[^ ^37^, ^38^ ^]^ In contrast peaks in the palladium‐silver alloy appear much broader than in the respective palladium or silver reference films. In fact, the peaks are so broad that they span almost the complete range of angles between the peaks observed in the Silver and Palladium reference films. However, the center of the peaks observed in the palladium‐silver alloy films are shifted towards the Palladium peak position. Peak positions between that of Palladium and Silver has previously been interpreted as evidence of alloy formation achieved by mechanochemical synthesis.^[^ ^39^ ^]^ Thus, the intermediate peak position in the current study points to the presence of a Palladium Silver alloy. However, a small peak corresponding to silver is observable too in the silver‐palladium films. Furthermore, as the higher energy side of the peaks observed in the silver‐palladium films extends beyond that of palladium, thus the presence of palladium within the palladium‐silver film is also likely. This is in line with the SEM morphology observation in section 3.2 that showed inhomogeneity in the films. Thus the presence of different phases as suggested by the XRD results is to be expected. An estimation of which intermetallic compounds are generated during the printing process is provided in the supporting information (Figure S2, Supporting Information).

For areas where a crystalline palladium‐silver alloy film is produced, the XRD peaks are expected to shift in proportion to the alloy ratio that has formed, here the ink was designed to produce a 79:21 palladium‐silver alloy.^[^ ^39^ ^]^ Thus, from Figure 6 it is apparent that some palladium‐silver alloy has formed upon heat treatment, as inferred from the peak center location of the alloy peak being present between the pure silver and pure palladium peaks. **Table** **3** shows the peak positions for silver, palladium and palladium‐silver alloy, from the positions and the alloy's peak shift from palladium and silver's peak values the alloy ratio can be calculated using the following equation:

(3) $$Ratio\;=\left(1-\frac{Mpeakposition-Alloypeakposition}{DifferencebetweenPdandAgpeak}\right)\times 100$$

Where M is the material we are calculating the ratio of. Table 3 shows the ratios calculated from these peaks.

**Table 3:** Peak locations and calculated ratios of Pd and Ag in the alloyed film for each XRD peak.

Peak	Pd	Ag	Pd‐Ag	Amount of Pd in alloy	Amount of Ag in alloy
111	40.20°	38.15°	39.78°	79.53	20.47
200	46.79°	46.28°	46.28°	79.37	20.63
220	68.19°	64.86°	67.52°	79.88	20.12

**Figure 7:**
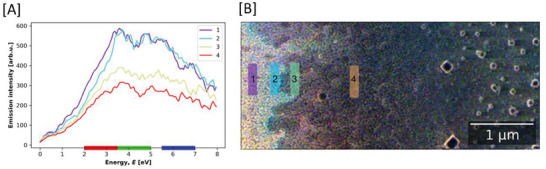
a) SE spectra from regions of inkjet printed PdAg, shown as masks in the color image in b). The color image b) is produced by assigning emissions to Red, Green and Blue color channels in the ranges 2.0–3.5 eV, 3.5–5.0 eV, and 5.5–7.0 eV respectively (ranges overlayed on energy axis in part a)). From Figure 7B is apparent that the inhomogeneity in composition appear on the nanoscale and might result in separate crystallites rather than continuous films.

The values obtained from the palladium‐silver alloy films, presented in Table 3, show that it is apparent that the film contains areas where an alloy ratio of 79:21 palladium‐silver has been achieved. The results also suggest that not all areas of the film have been successfully alloyed. Secondary Electron (SE) spectroscopy in combination with Secondary Electron Hyperspecteral Imaging (SEHI) was applied to extract data with high surface sensitivity and submicron spatial resolution relevant to the local film composition.^[^ ^40^, ^41^ ^]^ In contrast to conventionally applied energy‐dispersive X‐ray spectroscopy (EDX), which was performed and is presented in the Supporting Information (Figures S3 and S4, Supporting Information), SEHI has significantly enhanced surface sensitivity and spatial resolution.^[^ ^26^, ^28^, ^29^ ^]^ The enhanced surface sensitivity overcomes traditional sampling depth limitations encountered with EDX detecting x‐rays generated from bulk substrates rather than the thin PdAg films. The interaction depth and viable chemical information obtained from SEHI in comparison to that of EDX is highlighted in Monte Carlo (MC) simulations provided in the supporting information (Figure S5, Supporting Information). SEHI is therefore suited to optimize and deliver novel characterization insights that are outside the capability of conventional EDX. SEHI was applied in this study with resulting colored SEHI image is presented in **Figure** **7**. Reference SE spectra, which were used to determine the selection and identification of color RGB ranges, are presented in the supporting information S6. Although an overall composition of 79:21 Pd:Ag in the crystalline materials was determined from XRD (see Table 3) the color image of the inkjet‐printed PdAg film (Figure 7b) and derived SE spectra (Figure 7) reveal local variation in the relative metal contents. Including regions of Pd and Ag separation. Spectra were plotted from regions of the inkjet printed film, showing a palladium rich region (1), silver rich region (2) and mixed regions (3‐4) (Figure 7b).

### 3.3 Aerosol Jet Printed Palladium‐Silver Films

#### 3.3.1 Morphological and Chemical Characterization of Aerosol Jet Films

**Figure 8:**
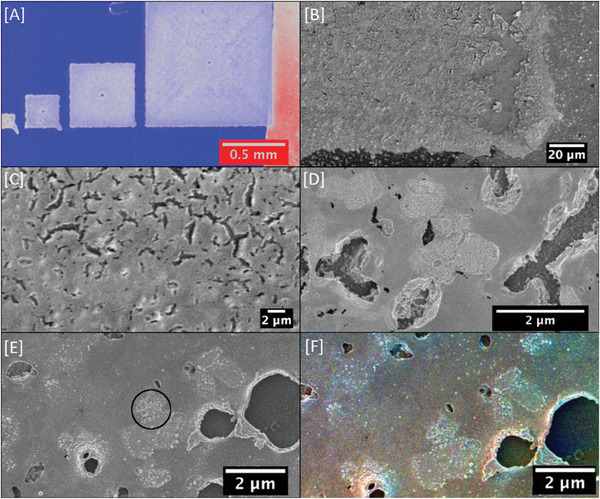
SEM and SEHI images of aerosol jet printed palladium‐silver as printed. a) is a false color image showing silicon substrate in red, PEDOT:PSS in blue and palladium‐silver in purple. b) is a high magnification area of the bottom right corner of the large palladium silver square. c) Further higher magnification image of printed large palladium‐silver square in b). d) Further higher magnification image of printed large palladium‐silver square in c). e) after hairline plasma treatment of 30s duration. f) SEHI image after plasma treatment. Red = Pd emission, Green = mixed emission, Blue = Ag emission. See SI for rationale.

**Figure 9:**
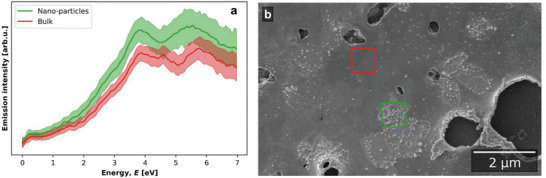
a) SE spectra plotted from bulk and nano‐particle dominated regions, outlined by the green and red boxes respectively in b), an image of the aerosol printed PdAg film.

Typical droplet sizes for aerosol jet printing are in the region of 1–5 µm^[^ ^42^ ^]^ a size expected to avoid the presence of large hollow structures in the deposited films. To avoid the potential for substrate crystallinity triggered phase separation, a buffer layer strategy was adopted. Poly(3,4‐ethylenedioxythiophene) polystyrene sulfonate (PEDOT:PSS), a widely available conducting polymer, was chosen as this buffer layer. The buffer was aerosol jet printed onto silicon, prior to the deposition of the palladium‐silver films. Palladium‐Silver squares of several sizes where printed with the largest square (in **Figure** **8**A) being 1×1 mm and the smallest 100×100 µm. False color imaging was used to visualize the different materials as follows: silicon substrate in red, PEDOT:PSS in blue and palladium‐silver in purple. To test the effect of the buffer layer on film print quality, an edge of a large palladium silver square was misaligned with that of PEDOT:PSS (shown in Figure 8B). Here an edge can be seen approximately in the center of the image delineating the extent of the PEDOT:PSS (black) buffer coating. Where printed over the buffer layer the palladium‐silver film was dense and smooth, whereas where printed directly on the silicon substrate it was observed to be delaminated over large areas. Examined at higher magnifications (Figure 8c,d) the palladium‐silver film printed on the PEDOT:PSS buffer it was revealed that cracks exist when moving towards the center of the printed square. Note that a perimeter film printing pattern was used to fill in the squares which does lead to differences in drying dynamics of the film across the square. The cracks are considered to be a consequence of differences in the films thickness at the center of the square. In the as‐printed films, bright patches were visible within smooth film areas. These patches tended to be in a near spherical shape and were on a length scale commensurate with previously reported aerosol jet droplet sizes.^[^ ^42^ ^]^ The as‐printed films were not stable with notable color changes over a timescale of hours. When imaged in the SEM in a stable condition (2 weeks after printing) and at high magnification the presence of individual nanoparticles at the rim of the bright patches (Figure 8E) is apparent. SEHI analysis and color representation (**Figure** **9**F) based on reference spectra suggest that at least some of the nanoparticles consist of Ag (blue color). Notwithstanding the scope for future optimization, optical images of complex array of interdigitated electrodes presented in Figure S7 (Supporting Information) show the capacity of aerosol jet printing to create fine printed lines and films. Such printing further demonstrates the flexibility of both deposition techniques over traditional coating methodologies for silver palladium catalysts. This is integral for the development of hydrogen separation devices as the creation of finely printed PdAg alloy lines can overcome embrittlement issues faced by purely Pd films. The precision with which patterns can be deposited precludes the need for masks reducing waste which is particularly advantageous when working with high cost materials.

Whilst the PEDOT:PSS buffer layer approach was proved to be important in obtaining well adhered films, it does not allow the use of the thermal annealing treatment that was used to convert the inkjet printed films into palladium‐silver alloys. Therefore, an atmospheric plasma “Hairline plasma” was used to explore if plasma annealing can be successfully performed on buffer protected substrates. When examined, defined nano‐particles assemblies are apparent in the former bright patches. SE spectra (**Figure** **9**) collected from such nano‐particle covered areas display the same characteristics of inkjet printed (and thermally annealed) palladium films (described in section 3.2.1). Whereas, the smooth bulk regions do not exhibit patches or nano‐particles, they do display consistent characteristics with that of palladium‐silver alloys. A systematic study considering variables such as filling pattern used during printing, the type and plasma treatment time could be used to optimize film morphology and chemical homogeneity but is beyond the scope of this study.

## 4 Conclusion

In this study a stable MOD ink containing palladium and silver has been developed and shown to be usable for ink jet and aerosol jet printing of films that may be subsequently processed into palladium‐silver alloy films using a thermal or plasma annealing process. The developed ink had a Z number of 6.48 and is well within the optimal range for ink and aerosol jet printing. Both jet‐printing methods formed films with complex morphology, with chemical variations from nano to the micron scale as revealed by SEHI. Although further optimization is still required to generate homogeneous films, inkjet printed and subsequently annealed (300 °C in inert atmosphere for 1 hour) films produced have an average alloy ratio of 79:21 palladium based on XRD analysis. The authors believe that these are the first example of inkjet printed palladium‐silver alloys. This study also provides evidence that films of palladium‐silver can be deposited by aerosol jet printing and plasma annealing, proposing a potential novel room temperature approach for the deposition of palladium‐silver MOD inks which were originally designed for deposition on heated substrates. An aerosol jet printing approach that does not requiring high temperature annealing holds the promise of printing Palladium‐Silver Alloys onto temperature sensitive polymer substrates. This approach would open the opportunity for producing polymer substate Palladium‐Silver films printed for use as catalysts for CO_2_ reduction in electrolyzer membranes.

## Conflict of Interest

The authors declare no conflict of interest.

## Supporting information

Supporting Information

## Data Availability

The data that support the findings of this study are openly available at 10.15131/shef.data.24798834.
