# Supplementary material for: Revealing The Morphology of Ink and Aerosol Jet Printed Palladium‐Silver Alloys Fabricated from Metal Organic Decomposition Inks
Source: Adv Sci (Weinh). 2023 Dec 25;11(10):2306561. doi: 10.1002/advs.202306561 (PMC10933619; doi:10.1002/advs.202306561)
Supplement: Supplementary file 1 — Supporting Information [file ADVS-11-2306561-s001.pdf]

ADVANCED SCIENCE
Open Access

Supporting Information

for *Adv. Sci.*, DOI 10.1002/advs.202306561

Revealing The Morphology of Ink and Aerosol Jet Printed Palladium-Silver Alloys Fabricated from Metal Organic Decomposition Inks

Nicholas T.H Farr, Matthew Davies, James Nohl, Kerry J. Abrams, Jan Schäfer, Yufeng Lai, Torsten Gerling, Nicola Stehling, Danielle Mehta, Jingqiong Zhang, Lyudmila Mihaylova, Jon R. Willmott, Kate Black and Cornelia Rodenburg*

Supporting Information: Revealing the morphology of Ink and Aerosol jet printed Palladium-Silver Alloys fabricated from Metal Organic Decomposition Inks

Nicholas T.H Farr[1,2], Mathew Davies[3], James Nohl[1]. Kerry J. Abrams[1], Jan Schäfer[4], Yufeng Lai[3], Torsten Gerling[4], Nicola Stehling[1], Danielle Mehta[5], Jingqiong Zhang[6], Lyudmila Mihaylova[6], Jon R. Willmott[3], Kate Black[5], Cornelia Rodenburg[1,2].

[1]Department of Materials Science and Engineering, Sir Robert Hadfield Building, Mappin Street University of Sheffield, Sheffield S1 3JD, UK.

[2]Insigneo Institute for In Silico Medicine, The Pam Liversidge Building, Sheffield, UK

[3]Department of Electronic and Electrical Engineering, Portobello Centre, Pitt Street. University of Sheffield, Sheffield, S1 4ET, UK.

[4]Leibniz Institute for Plasma Science and Technology e.V. (INP), Felix-Hausdorff-Str. 2, 17489 Greifswald, Germany.

[5]School of Engineering, University of Liverpool, Liverpool, L69 3BX UK.

[6]Department of Automatic Control and Systems Engineering, The University of Sheffield, Amy Johnson Building, Portobello Street, Sheffield, S1 3JD, UK.

1. Additional Methods

1.1. Energy dispersive X-ray spectroscopy (EDX)

FEI Helios Nanolab G3 (FEI Company, USA) SEM equipped with an Energy Dispersive X-ray Spectroscopy (EDX) detector (Oxford Instruments, UK) was used to capture EDX maps and spectra. The EDX spectra and maps were obtained with a 15 keV accelerating voltage using a 13 nA probe current at a working distance of 4 mm. Data analysis was automated by the application of Aztec EDS analysis software (Oxford Instruments, UK).

2. Additionally Results

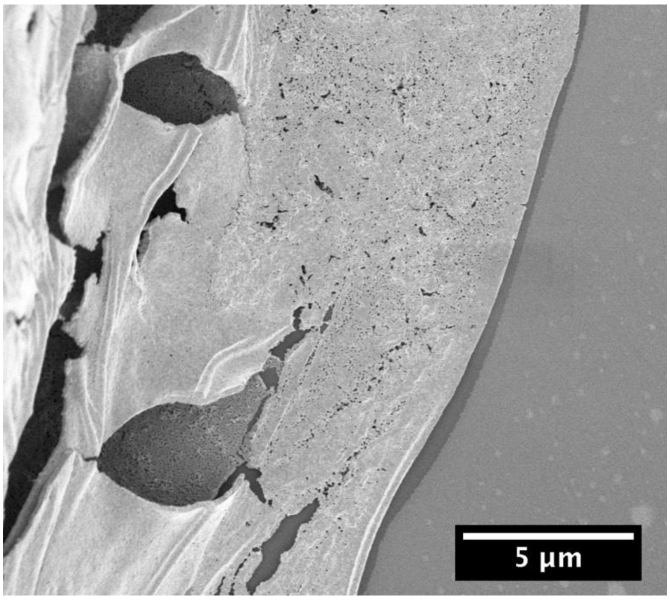

Figure S1: LV-SEM of five pass Palladium film with sheet like structures on the left (centre of ink droplet remnant) that transitions to porous largely 2D networks of nanoparticles at the edges of the former droplet. Holes in the sheet structures reveal that such 2D networks are also formed underneath the sheets. This confirms the critical roll of the substrate to convert ink into nanoparticles during the thermal annealing step.

Figure S2A presents the XRD signatures of Pd, Ag and PdAg films that were deposited from MOD inks in 10 passes. To capture an estimation of which intermetallic compounds are generated during the printing process Figure S2 contains an inset of previously published XRD signatures of pure Au and Pd, and Au(3)(1), Au(1) Pd(1), and Au(1)Pd(3) alloys [1]. Comparison of these signatures with that of the XRD obtained from the PdAg film characterised in this study, shows that Au(1)Pd(3) as a possible intermetallic compound present. However, as the reference peaks presented are broad in nature this cannot be stated with certainty and future work will need to confirm this finding as well as consider the presence of localised intermetallic compounds.

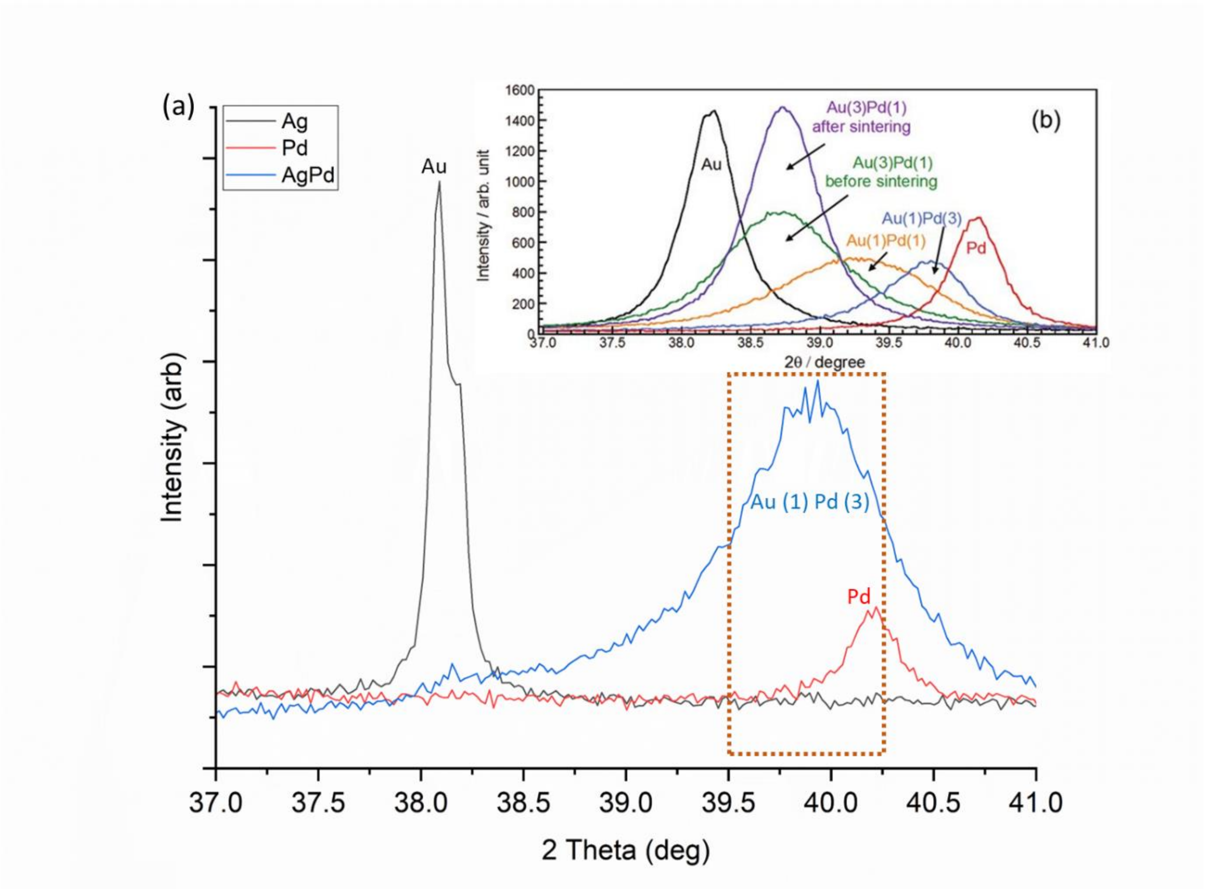

Figure S2: [A] XRD of Pd, Ag and Pd-Ag films printed onto glass and heat treated at 300°C for 1 hour in a nitrogen atmosphere. [B] previously published XRD signatures of pure Au and Pd, and Au(3)(1), Au(1) Pd(1), and Au(1)Pd(3) alloys. Reprinted with permission from [1].

Table S1 Hairline Plasma Source

Table S1 shows parameters of the plasma source used in this work

Source	Hairline Plasma
Geometry	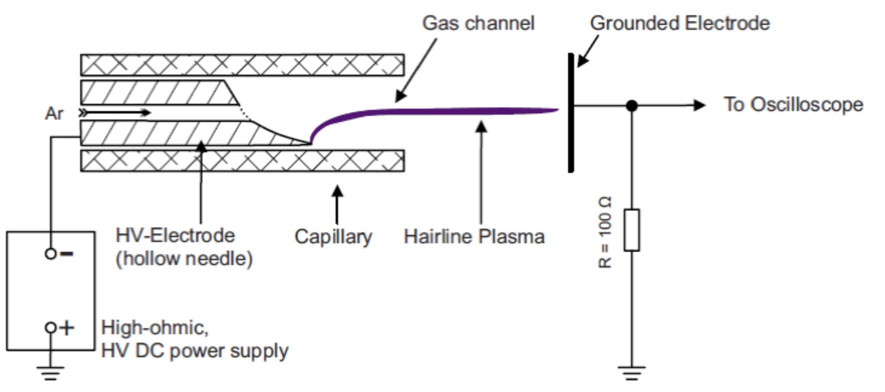
Characteristic diameter	1 mm (capillary)<br>0.1 mm (filamentary discharge)
Reactive gas	Argon
Flow rate	0.5 slm
Excitation frequency	1-3 kHz self pulsed (FWHM 10 ns)
Power	0.1 – 0.5 W

Figure S3 presents energy-dispersive X-ray spectroscopy (EDX) data collected from both PdAd Inkjet printed film (10 pass) and PdAd aerosoljet printed films. Figure S3A displays an SEM image of the PdAd Inkjet printed film (10 pass) with two ROI marked, I and II with accompanying EDX spectra. For both ROI`s a prominent Si peak was noted, with Pd and Ag emission obtainable at low counts. As highlighted in the main text EDX is not considered an accurate technique for shallow surface characterisation. The sampling depth of EDX is typically in the scale of a few microns, therefore, for thin films with a thickness less than a few microns the majority of EDX emission is expected to originate from the bulk substrate (in this instance Silicon). It is possible to decrease the sampling depth of EDX by reducing the incident beam`s accelerating voltage, however this can result in the reduction of the amount of X-Ray counts and provide unreliable data. This is of particular concern for this study as both Ag and Pd emitted X-rays of similar energies levels. Despite these expected

limitations EDX did yield elemental maps for Si, Ag, and Pd (Fig S3. B-D) for the PdAd Inkjet printed film. These maps confirmed the presence of Ag and Pd within the film. The EDX also highlighted areas of poor film coverage consisting of holes with strong Si emission. Figure S3 also presents EDX data collected from the PdAd aerosoljet printed film.

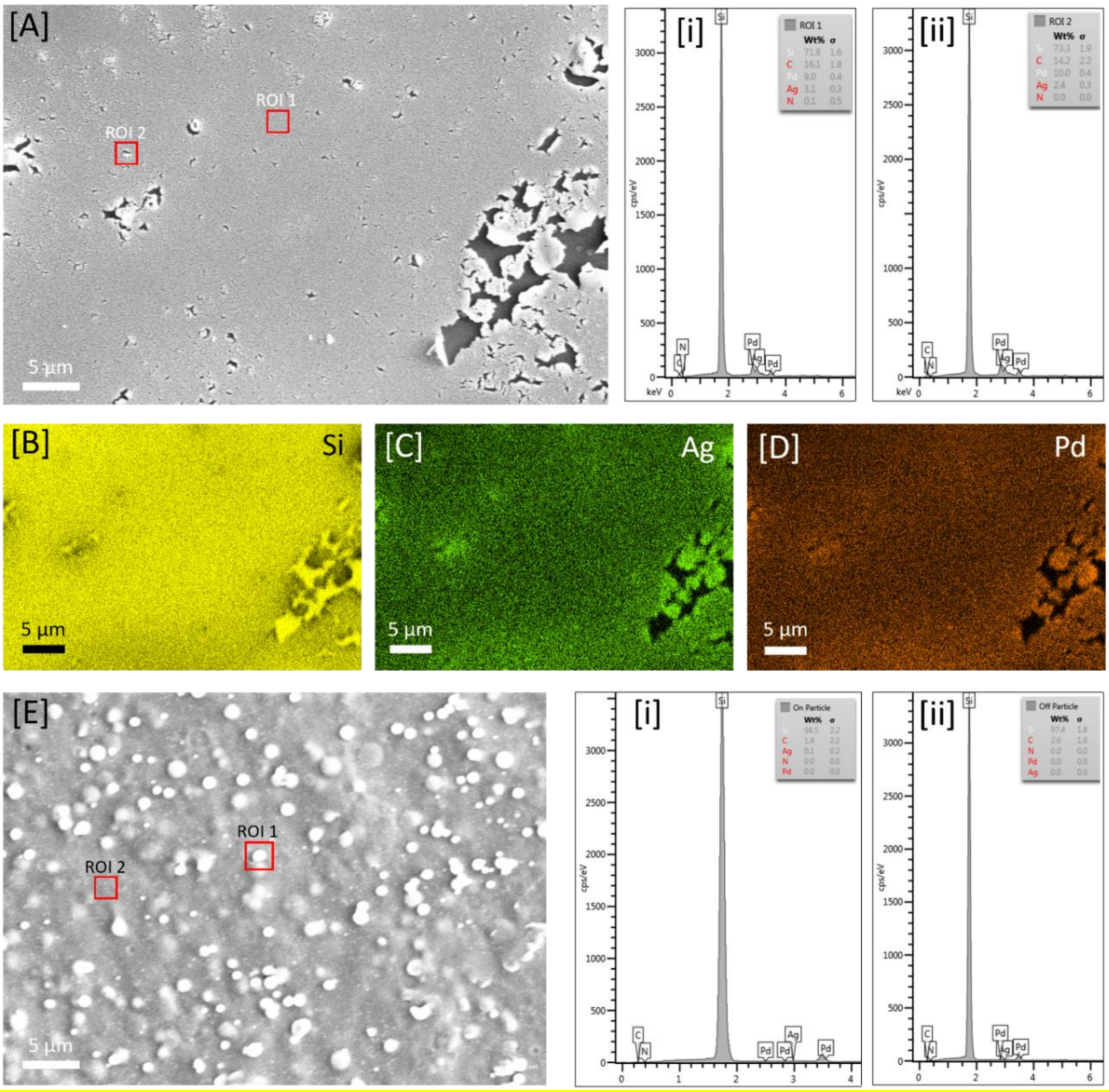

Figure S3 - (a) SEM image of PdAd Inkjet printed film (10 pass) with two ROI marked. I and II display the accompanying EDX spectra for the ROIs. [B] presents the EDX map of Silicon for the PdAd Inkjet printed film (10 pass). [C] presents the EDX map of Silver for the PdAd Inkjet printed film (10 pass). [D] presents the EDX map of Palladium for the PdAd Inkjet printed film (10 pass). (E) SEM image of PdAd aerosoljet printed film with two ROI marked. I and II display the accompanying EDX spectra for the ROIs.

Figure S3E shows an SEM image of the PdAd film with two ROI marked, I and II (labeled in figure S3E) with accompanying EDX spectra. For both ROI`s a prominent Si peak was noted, similar to that of the InkJet printed film, however for this film no observable traces of Pd and Ag emission were obtainable. As a result of the lack of X-Ray counts related to Ag or Pd no EDX maps could be obtained for the aerosoljet printed film.

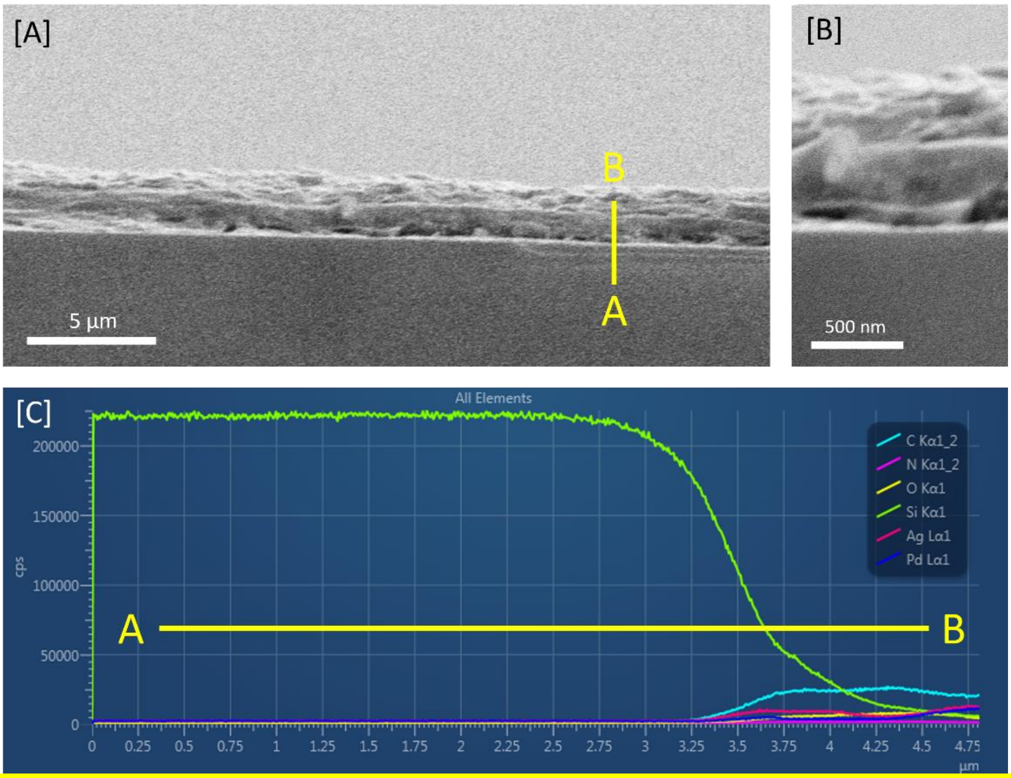

Figure S4 - (a-b) SEM images of cross sections of the PdAd aerosoljet printed film. (c) presents a EDX lines scan of the cross-section from marked points A – B.

It is therefore hypothesised that the thickness of the aerosoljet printed film was less than that of the sampling depth of EDX. To confirm this a cross-section of the aerosoljet printed film was prepared, imaged (Fig S4. A&B) and an EDX line scan was performed (Fig S4. C). The EDX line scan confirmed the presence of Ag and Pd within the aerosoljet printed film. The line scan also identified the thickness of the AgPd film to be around > 300 nm. This was measured at the point at which the Si emission (from the substrate) became dominate over that of the Ag and Pd.

To better understand the interaction depth and viable chemical information that was obtained from SEHI compared to that of EDX a Monte Carlo (MC) model [2] was established which was applied to simulate the electron trajectory of the different primary beams. The results of the simulations are presented in Figure S5, showing MC simulations of the interaction of 400 primary electrons at 1 kV, 10 kV, 15 kV and 20 keV with a simulated PdAg film (including a bulk silicon substrate). The resulting electron emission trajectories are coloured coded by interaction between PdAg (blue) and Silicon (Green). In contrast to EDX, which typically employs a primary beam accelerating voltage of between 10 – 20 KeV (required to generate reliable x-ray counts as reducing the beam voltage from 20 kV to 2 kV can produce 35x less X-ray intensity [3]), SEHI is preformed using lower KeV range. In the instance of this study an accelerating voltage of 1 kV accelerating voltage was selected for SEHI. As depicted in Figure S5, a 1 kV interacts entirely with the Ag/Pd surface film whereas higher

accelerating voltages interact significantly with the underlying bulk silicon substrate. As described in depth in previous studies [4,5], SEHI generates its chemical information through the scattering of secondary electrons (SEs). SEs are produced throughout the primary electron's interaction depth, but only SEs produced within the SE escape depth can be detected, making SEHI sampling very surface sensitive.

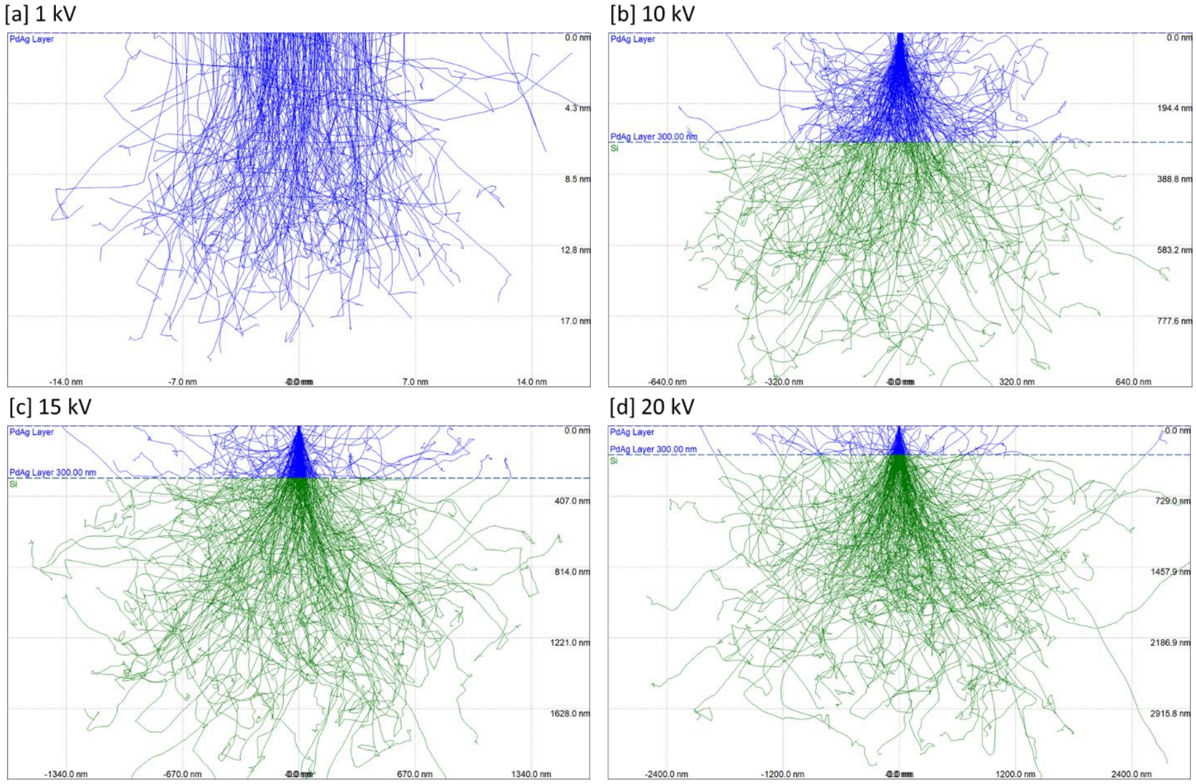

Figure S5 - Monte Carlo simulation derived data [2] depicting the interaction 400 primary electrons at (a) 1 kV, (b) 10 kV (c) 15 keV and (d) 20 kV with simulated PdAg film (containing carbon as identified through EDX measurements) with a silicon substrate. The resulting of electron emission trajectories are coloured by interaction between PdAg (blue) and silicon (Green).

Figure S6 presents SE energy ranges of interest for the PdAg printed films which were identified by taking measurements from Ag and Pd reference materials. The reference material spectra are plotted in Figure S6A. Regions of the hyperspectral data volume that spectra are plotted from are shown in Figure S6 A and B. Flatter regions of the reference material were chosen in order to reduce the influence of topology. The Red, Green and Blue ranges (2.0-3.5 eV, 3.5-5.0 eV and 5.5-7.0 eV respectively) on the energy axis of Figure S6 were identified to roughly indicate Red: Pd dominated emissions, Green: Ag and Pd mixed emissions and Blue: silver dominated emissions. Absolute SE

peak positions can vary dependent on the detector/apparatus as the spectrum may shift by 1 eV (+/-) [6].

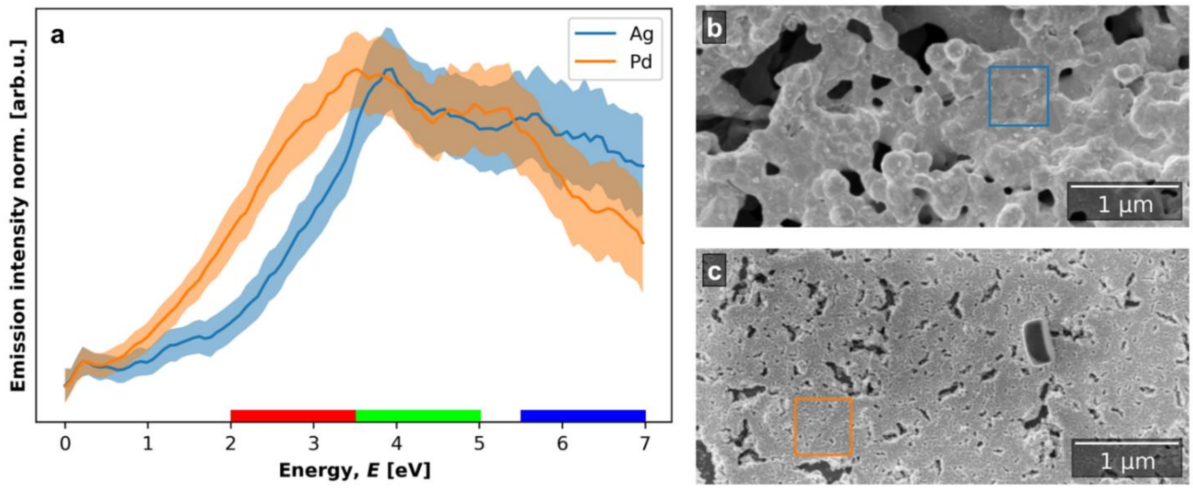

Figure S6 - (a) SE spectra from metallic silver (Ag) and palladium (Pd) references. Spectra are normalised to the maximum of the average spectrum (solid line). The shaded area is the standard deviation of spectra plotted at each pixel position in the region of interest (ROI). Images of (b) Silver (Ag) and (c) palladium (Pd) reference materials with ROIs from which pixel location spectra were derived.

Figure S7 presents complex array of interdigitated electrodes printed using a silver nanoparticle ink (Sigma Aldrich silver dispersion 798738) forming electrodes for a liquid crystal spatial light modulator. The circular region was pixelated in order to selectively alter the transmission of a laser beam to improve optical communications.

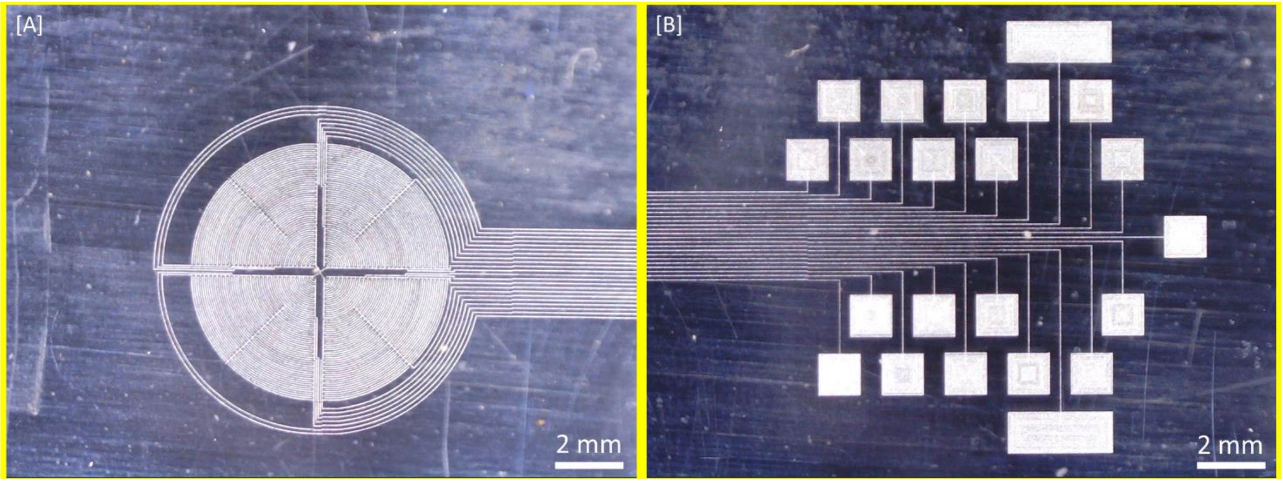

Figure S7: Optical images of interdigitated electrodes aerosol jet printed using a silver nanoparticle ink.

References

1. Tsuji M, Uto K, Hayashi J, Yoshiwara A. Synthesis of Flower-Like AuPd@SiO2 Nanoparticles with a Broad Light Extinction for Application to Efficient Dye-Sensitized Solar Cells. Part. Part. Syst. Charact. 2018. 35. 1700396.
2. Demers H, Poirier-Demers N, Couture AR, et al. Three-dimensional electron microscopy simulationwith the CASINO Monte Carlo software. Scanning.52011;33(3):135–146
3. Burgess S., Sagar J., Holland J., Li X., Bauer F.. Ultra-low kV EDS – a new approach to improved spatial resolution, surface sensitivity, and light element compositional imaging and analysis in the SEM. Microsc. Today, 25 (2) (2017), pp. 20-29,
4. Nohl J.F. Secondary electron hyperspectral imaging: nanostructure and chemical analysis for the LV-SEM. Mat. Sci. Technol., 36 (5) (2020), pp. 527-539.
5. Nohl J.F, Farr N., Sun Y., Hughes G., Cussen S., Rodenburg. C., Low-voltage SEM of air-sensitive powders: From sample preparation to micro/nano analysis with secondary electron hyperspectral imaging, Micron, Volume 156, 2022, 103234, ISSN 0968-4328, https://doi.org/10.1016/j.micron.2022.103234.
6. Lang, B. Tatarenko, S. Discrete levels in the energy distribution of secondary electrons emitted by palladium crystals. *Solid State Commun*. 1979, 31, 303.
